# Assessing species-specific neonicotinoid toxicity using cross-species chimeric nicotinic acetylcholine receptors in a *Drosophila* model

**DOI:** 10.1038/s41598-025-20109-3

**Published:** 2025-10-16

**Authors:** Anna Lassota, James J. L. Hodge, Matthias Soller

**Affiliations:** 1 School of Biosciences, College of Life and Environmental Sciences, University of Birmingham, Edgbaston, Birmingham, B15 2TT UK; 2 School of Physiology, Pharmacology and Neuroscience, Biomedical Sciences Building, University of Bristol, University Walk, Bristol, BS8 1TD UK; 3 Division of Molecular and Cellular Function, School of Biological Sciences, University of Manchester, Oxford Road, Manchester, M13 9PT UK

**Keywords:** nAChR, Neonicotinoids, *Apis mellifera*, *Drosophila*, CRISPR-Cas9 genome engineering, Pesticides, Mutagenesis, Evolutionary biology, Ion channels in the nervous system

## Abstract

Nicotinic acetylcholine receptors (nAChRs) are ligand-gated ion channels and the main mediators of synaptic neurotransmission in the insect brain. In insects, nAChRs are pivotal for sensory processing, cognition and motor control, and are the primary target of neonicotinoid insecticides. Neonicotinoids are potent neurotoxins, and pollinators such as honey bees are more sensitive and affected by extremely low sub-lethal doses. nAChR subtypes exist as homomers of α-subunits or heteromers composed of α and β subunits. The honey bee nAChRα8 subunit is orthologous to nAChRβ2 in *Drosophila*, raising the question of whether this α to β change makes flies less sensitive to neonicotinoids. To investigate species-specific aspects of neonicotinoid toxicity, we CRISPR-Cas9 engineered a cross-species chimeric nAChR subunit by swapping the ligand-binding domain in *Drosophila* of nAChRβ2 with honey bee nAChRα8. Phenotypic assessment revealed significantly impaired motor functions in climbing and flight assays when comparing flies carrying the α8/β2 chimeric channel to wild type or a β2 knock-out. Despite these motor deficits, both flies expressing the α8/β2 chimeric receptor and β2 knock-out flies showed significantly increased survival after exposure to neonicotinoids thiamethoxam and clothianidin, compared to wild type flies. Combinatorial exposure to different insecticides did not reveal differences. These findings highlight the critical role of nAChR subunit composition in motor control, and demonstrate how subtle structural modifications within a single nAChR subunit can profoundly impact motor function and pesticide response, offering new insights into the molecular mechanisms of neurotoxicity across species.

**Supplementary Information:**

The online version contains supplementary material available at 10.1038/s41598-025-20109-3.

## Introduction

Pesticides are indispensable for protecting global agricultural yields by reducing crop losses caused by pests^1^. Among these, neonicotinoids are highly effective due to their broad-spectrum efficacy and systemic activity, which enable integration into plant tissues for targeted pest control, and versatility in application methods, such as seed coating, to reduce environmental contamination^2,3^. Neonicotinoids exhibit high toxicity to insects due to their selective action on insect nAChRs. While originally developed as safer alternatives for vertebrates, accumulating evidence has raised concerns about their effects on mammals, including humans^4–8^. Furthermore, neonicotinoids do not distinguish between pests and other insects, and harm beneficial pollinators like honey bees, which are critical for both wild flora and agricultural crops^9–11^. The widespread decline of pollinators, partially attributed to pesticide exposure, poses significant ecological and economic challenges, highlighting the need for pest-control strategies with reduced non-target effects.

Honey bees, wild bees and other pollinators are adversely affected by sub-lethal doses of neonicotinoids (2 ng/ml compared to the LC50 dose of 4.28 µg/ml) impairing foraging behaviour, cognition, navigation, weakening of the immune system, and reducing reproductive success and genetic diversity within a colony^9,12–23^. Neonicotinoids, which target cholinergic neurotransmission, inhibit mushroom body Kenyon cell activity^24^ and disrupt memory and olfactory sensory neuron activity in pollinators, including bumblebees and honey bees (*Apis mellifera*), as well as fruit flies (*Drosophila melanogaster*)^24–27^.

The sensitivity of insects to neonicotinoids varies considerably between species. For example, the 24-h 50% lethal concentration (LC50) of thiamethoxam (TMX) is 3.13 µg/ml for adult *Drosophila*^28^, whereas for honey bees it is 4.28 µg/ml^23^. Variation arises from differences in the repertoire of cytochrome P450 detoxification genes. *Drosophila* encodes 85 of these neonicotinoid-metabolising enzymes, whereas honey bees have only 46^29,30^. Additionally, honey bees have fewer antioxidant gene paralogs than *Drosophila*, which has an expanded antioxidant defence system. Since neonicotinoids can induce oxidative stress, weaker antioxidant capacity may contribute to the greater sensitivity^31^.

**Fig. 1:**
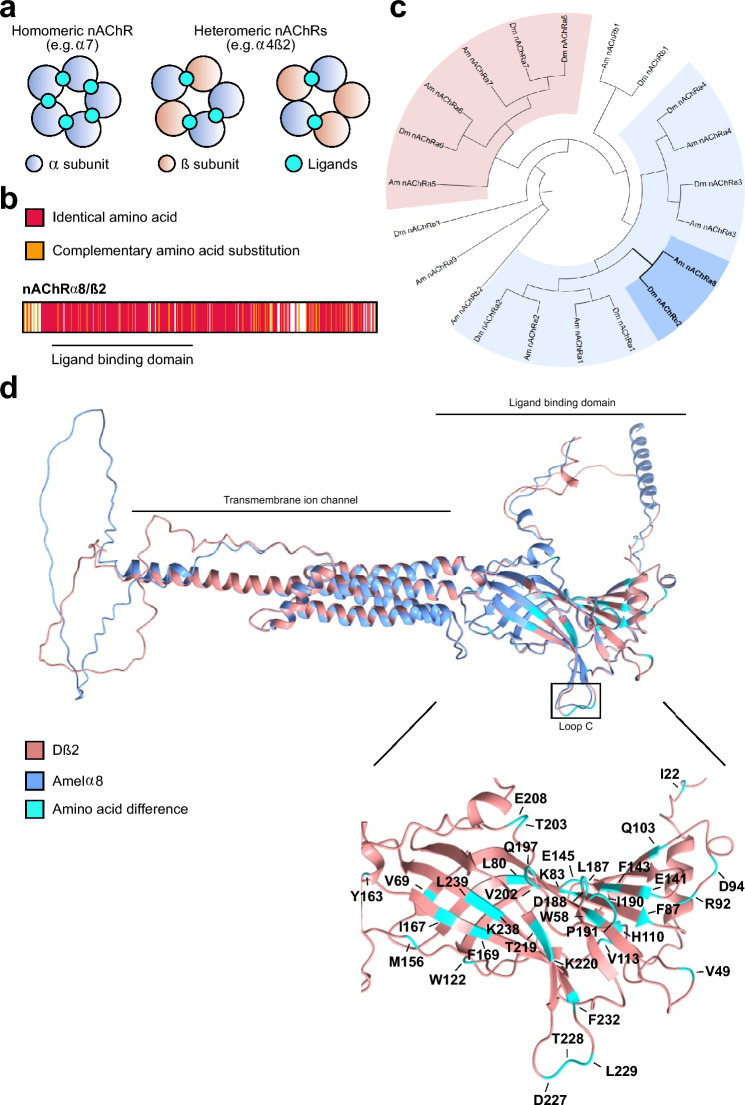
Highly conserved *Drosophila* β2 subunit originated from an α subunit in other insects. (**a**) Conceptualised schematic of insect nAChR receptors illustrating possible examples of α–α and α–β homo- and heteropentameric assemblies (α: blue, β: pink). Cyan coloured circles indicate potential ligand-binding sites, which form at α–α or α–β interfaces depending on subunit arrangement. (**b**) Amino acid alignment of Dβ2 and honey bee Amelα8 subunits. Identical amino acids are marked in red, complementary amino acid substitutions are labelled in orange, and non-complementary amino acid substitutions are unmarked. The ligand binding domain is underlined. (**c**) Molecular phylogenetic analysis of all nAChR subunits present in *Drosophila* and honey bee with two major α subunits clades highlighted with light blue and pink (subunits highly conserved with mammalian α7). Dβ2 and Amelα8 (bold) clade is highlighted in darker blue. Species codes are Dm: *D. melanogaster,* Amel: *A. mellifera*. (**d**) Structural alignment of *Drosophila* Dβ2 (blue) and honey bee Amelα8 (pink) with all amino acid differences in *D. melanogaster* ligand binding domain indicated (cyan).

Another reason contributing to this species-specific susceptibility could be the composition of nicotinic acetylcholine receptors (nAChRs). nAChRs are essential for neurotransmission, synaptic plasticity, and neuronal development, making them prominent targets for neonicotinoid pesticides^32–35^. These pentameric cys-loop ligand-gated cation channels consist of α and β subunits forming homo- or hetero-pentamers. Ligand binding pockets, located at α-α or α-β interfaces (Fig. 1a), consist of loops A, B, and C from an α subunit and loops D, E, and F from the adjacent α or β subunit^36–38^. Importantly, β subunits lack the ability to form functional homo-pentamers due to the absence of loops A, B, and C^36,38^. Consequently, nAChR subunit composition affects their properties and determines susceptibility to agonists, such as acetylcholine or neonicotinoids. Although homo-pentamers potentially can bind five ligands, binding of one ligand in human α7 pentamer is sufficient to induce the full response^39^.

Compared to vertebrates, the majority of insects have smaller nAChR gene families^40–42^. *D. melanogaster* encodes 10 subunits (Dα1-Dα7 and Dβ1-Dβ3), the honey bee encodes 11 (Amelα1-Amelα9 and Amelβ1-Amelβ2)^43^, and the cockroach *Periplaneta americana* has 19^44^. These subunits are classified as α or β based on the presence or absence of two adjacent cysteines in loop C of the ligand binding domain (LBD) which are crucial for ligand interaction^45^.

Insect nAChR genes include both highly conserved and more divergent members^40,41^. Among these conserved subunits is the nAChRα8 present in many insects but notably absent in certain Dipterans, including *D. melanogaster*^41^. Instead, *Drosophila* encodes the Dβ2 subunit, which exhibits 75% overall sequence identity to the Amelα8 subunit, with striking 92% identity within the LBD (Fig. 1b and Supplementary Fig. 1). Notably, the absence of loop C in Dβ2 which is essential for ion channel gating, highlights a structural divergence that may contribute to species-specific functional properties and differences in neonicotinoid susceptibility^46,47^. Further, specific amino acid substitutions in nAChR subunits are known to impact insecticide susceptibility. For instance, *Drosophila* mutants lacking the *Dβ1* gene or carrying the variant R81T in the Dβ1 subunit originally found in aphids exhibit about 10 and 100-fold increase in resistance to clothianidin and thiamethoxam, respectively^48–50^.

Here, we examine the phylogenetic relationship and functional implications of nAChR subunits in *Drosophila melanogaster* and *Apis mellifera*. Phylogenetic analysis confirms that Dβ2 and Amelα8 are orthologous, suggesting a conserved ancestral origin despite their classification as β and α subunits, respectively (Fig. 1c). To investigate whether this α-to-β subunit divergence contributes to neonicotinoids sensitivity, we employed CRISPR-Cas9 gene-editing technology to generate chimeric *Drosophila* flies expressing a chimeric nAChR receptor. Specifically, we replaced the LBD of the Dβ2 subunit with that of the Amelα8 subunit, providing a model to explore cross-species receptor functionality. Behavioural and survival assays revealed significant impairments in motor functions and altered sensitivity to insecticides, highlighting how subtle structural differences within the ligand-binding domain of a single nAChR subunit can influence pesticide response. Our results provide insights into the evolutionary trajectories of nAChR subunits and their roles in mediating neurotoxic compound interactions, establishing a valuable *Drosophila* model for investigating cross-species pesticide toxicity.

## Results

### Species-specific structural differences in the ligand-binding domains of *Drosophila* β2 and honey bee α8 nAChR subunits

To investigate whether Dβ2 represents a divergent lineage from other β subunits or shares a closer evolutionary relationship with α subunits, we used Alphafold structural modelling. Our analysis confirms overall structural conservation between Dβ2 and Amelα8, particularly in the LBD, with the exception of the loop C, which is absent in Dβ2 (Fig. 1d)^51^.

### *nAChR* subunits exhibit developmental dynamics and cell-specific distribution

**Fig. 2:**
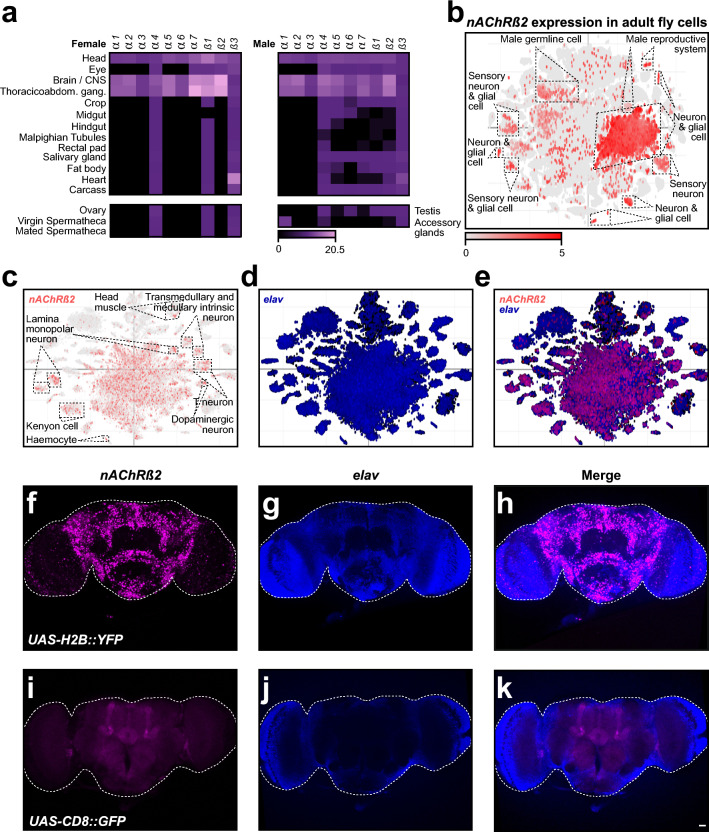
*Drosophila nAChRβ2* is highly expressed in adult *Drosophila* CNS but not in every cell. (**a**) Expression levels of all *Drosophila nAChR* subunits across various adult tissues in males (left) and females (right) obtained from FlyAtlas 2. Receptor subunits are indicated on top and tissues on the left. The colour scale bar at the bottom indicates transcript abundance in fragments per kilobase of transcripts per million mapped reads (FPKM). (**b**) Single-cell RNA-seq visualisation of *nAChRβ2* gene expression from ASAP in whole adult fly showing in red expression of *Dβ2* on top of all cells shown in grey. Selected annotated cell clusters are indicated. The colour scale bar at the bottom indicates transcript abundance in fragments per kilobase of transcripts per million mapped reads (FPKM). (**c**–**e**) Single-cell RNA-seq visualisation maps showing qualitative expression of *nAChRβ2* (red, c) on top of all cells (grey, c), the panneuronal marker *elav* (blue, d) or both (e) on top of all cells (grey) in the adult fly heads from ASAP. Selected annotated cell clusters are indicated. (**f**–**k**) Expression of *nAChRβ2* visualised with a *GAL4* inserted in the endogenous locus in adult *Drosophila* brains using *UAS* nuclear localised YFP reporter (f–h) or using *UAS* cell-membrane associated GFP reporter (i-k). *Dβ2* expression (f, i) overlaps with the neuronal marker *elav* (g, j), as shown on merged pictures (h, k), confirming *Dβ2* expression in neuronal populations. Scale bar is 20 µm.

To investigate the expression patterns of *nAChR* subunits, we analysed tissue specific expression data in adults, taking into account enrichment values providing the gene abundance measure in a tissue relative to that in the whole fly^52^. The majority of *nAChR* subunits exhibits higher expression levels in females than in males (Fig. 2a). In both sexes, *nAChR* subunits are predominantly expressed in the central nervous system (CNS) and thoraco-abdominal ganglion, with *Dβ2* being the most highly expressed subunit in the brain. In other tissues, however, the levels of expression differ between sexes, with *Dβ2* and *Dα7* being the most expressed subunits in the thoraco-abdominal ganglion of males and females, respectively. Furthermore, only two subunits, *Dα4* and *Dβ3*, are expressed across all tissues, with *Dβ3* showing notable expression in the heart. Specific differences were observed between sexes. For instance, in the male salivary glands, carcass, and crop, only *Dα1*, *Dα2*, and *Dα3* are expressed, while *Dα4*, *Dβ1*, and *Dβ3* are restricted to females.

Further, we investigated whether the expression of *nAChR* subunit changes during development. Larval tissue analysis revealed that *Dα3* is not expressed in any tissue (Supplementary Fig. 2a), suggesting it is dispensable during development. In contrast, *Dβ3* is expressed in all larval tissues, with the garland cells showing the highest expression. The larval CNS is the only structure expressing all *nAChR* subunits, with *Dβ3* showing the highest levels. Overall, *nAChR* subunit expression is relatively low in most tissues in both larvae and adults (Fig. 2a and Supplementary Fig. 2a).

To refine the spatial expression of *β2* in *Drosophila* cells in more detail, we analysed expression at single-cell resolution^53^. In whole adult flies, *Dβ2* was found to be expressed in various cell types, including neurons, sensory neurons, glial cells, as well as male germline and reproductive system, however, its expression is not ubiquitous (Fig. 2b). Notably, a prominent cluster of neurons classified as adult fly head CNS cells strongly expressed *Dβ2*, suggesting it may have a significant role in neuronal circuits^26,54^. Since *Dβ2* displayed the highest expression levels in the CNS, we further analysed single cell expression data for the larval brain and whole adult fly head. In the adult brain, *Dβ2* expression was observed in distinct neuronal populations, including Kenyon cells, dopaminergic neurons or haemocytes, contrasting with the broader expression of the neuronal marker *elav* (Fig. 2c–e)^55^. In contrast, larval *Dβ2* was colocalised with *elav* in the majority of CNS neurons (Supplementary Fig. 2b–d)^56^.

To validate the expression patterns, we used *nAChRβ2*^*[2A-GAL4]*^ allele which has a *T2A-GAL4* sequence fused at the 3′ end of the gene, resulting in GAL4 expression as a separate protein under the control of *nAChRβ2* regulation^57^. Therefore, we analysed both larval and adult brains using nuclear *UAS-Histone2B::YFP* or membrane-bound *UAS-mCD8::GFP* reporters, co-staining neurons with anti-*elav* antibody. Consistent with our observations from the single cell expression analyses, *Dβ2* expression was restricted to specific neuronal subtypes, confirming its non-ubiquitous distribution (Fig. 2f–k and Supplementary Fig. 2e–j). Furthermore, we noticed enhanced *Dβ2* expression in Kenyon cells of the mushroom body and the ellipsoid body of the central complex, particularly when a membrane-bound GFP reporter was used (Fig. 2i–k)^54^.

Together, these findings reveal that nAChR subunit expression is dynamically regulated during development and exhibits sex- and cell-specific specialisation, highlighting the functional diversity of these receptors in neural circuits and other tissues. Furthermore, the observed developmental shift in *Dβ2* expression, from widespread larval CNS expression to more specialised adult neuronal populations, reflects the transition from simpler larval neural circuits to the more specialised and complex adult CNS.

### Chimeric *Amelα8/Dβ2* ligand-binding domain flies are viable with impaired motor functions

**Fig. 3:**
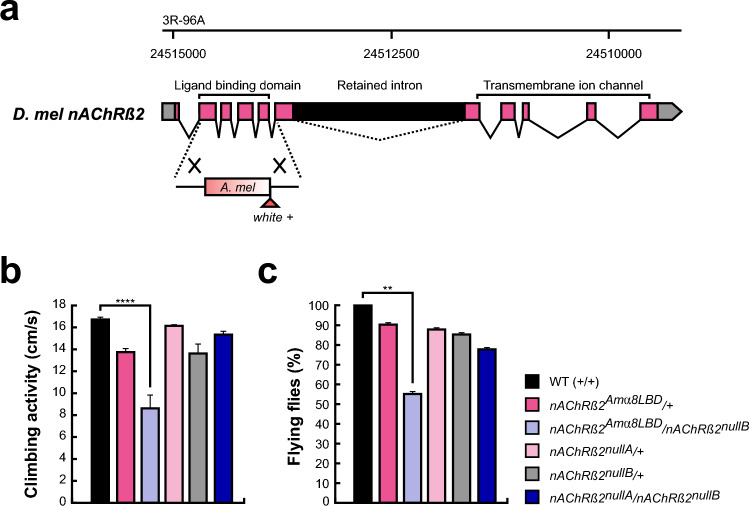
Chimeric *nAChRα8β2* flies exhibit locomotor defects. (**a**) Schematic of the endogenous *Dβ2* locus showing CRISPR-mediated replacement of the native ligand binding domain with the corresponding region from *A. mellifera nAChRα8* and the *white* + (*w* +) selection marker. Coding exons are marked in pink. The *w* + marker and bee LBD sequence were inserted through homology-directed repair, disrupting the reading frame and generating a *nAChRβ2* null allele. The reading frame can be restored by excising the PiggyBac transposon. (b) Climbing activity was assessed by negative geotaxis and is shown as means with the standard error from four biologically independent groups of 20 flies. Statistically significant differences are indicated by asterisks (****p = 7.5*10^–7^). (**c**) Flies of the indicated genotypes were tested for their flight ability shown as means with the standard error from four biologically independent groups of 10 flies. Statistically significant differences are indicated by asterisks (**p = 0.0066).

Since the two adjacent cysteines in the loop C are essential for ligand binding in nAChRs, we hypothesised that this difference may influence neonicotinoid susceptibility, making honey bees more vulnerable. Therefore, we generated chimeric *nAChRβ2/α8* (*nAChRβ2*^*Amα8LBD*^) flies, where the *β2* LBD coding region in *Drosophila* was swapped with the *Amelα8* LBD coding sequence using CRISPR-Cas9 genome engineering (Fig. 3a and Supplementary Fig. 3). Successful recombinant flies were marked with *white* + selection marker, disrupting the LBD, therefore resulting in a null allele (*nAChRβ2*^*nullA*^). The marker was then excised by PiggyBac transposase to restore the open reading frame and generate flies expressing the chimeric *nAChRβ2/α8* subunit (*nAChRβ2*^*Amα8LBD*^).

To normalise the genetic background, we used trans-heterozygotic flies for the *nAChRβ2*^*attP*^ allele (*nAChRβ2*^*nullB*^) in which the coding region was replaced with an *attP* site, *3xP3-RFP* and a *loxP* site^57^, together with the *nAChRβ2*^*nullA*^ allele. The chimeric *nAChRβ2*^*Amα8LBD*^ mutants, as well as *nAChRβ2*^*nullA*^ and *nAChRβ2*^*nullB*^ flies are fully viable when crossed with a chromosomal deficiency (*n* = 453, 275, 421, respectively).

To assess motor abilities, we performed negative geotaxis assays (Fig. 3b) and evaluated flight ability (Fig. 3c). In negative geotaxis assays the climbing ability of LBD swap mutant (*nAChRβ2*^*Amα8LBD*^/*nAChRβ2*^*nullB*^) was greatly reduced (*n* = 4 from 20 flies each, *p* = 7.5*10^–7^) (Fig. 3b) compared to wild type (WT). We did not detect significant differences in the *Dβ2* null mutant (*nAChRβ2*^*nullA*^/*nAChRβ2*^*nullB*^) nor the heterozygous mutant controls.

In flying ability assays, we observed the same, namely the LBD swap mutants (*nAChRβ2*^*Amα8LBD*^/*nAChRβ2*^*nullB*^) were impaired (*n* = 4 from 10 flies each, *p* = 0.0066) (Fig. 3c) compared to control flies, but not the *β2* null mutant (*nAChRβ2*^*nullA*^/*nAChRβ2*^*nullB*^) or heterozygote mutant controls.

### Chimeric *Amelα8/Dβ2* ligand-binding domain flies display resistance to insecticides

To investigate the impact of substituting the Dβ2 LBD with the honey bee Amelα8 LBD on neonicotinoid susceptibility, we selected two well-characterised neonicotinoids thiamethoxam (TMX) and clothianidin (CLO) known for their high toxicity to pollinators^7,50^.

TMX is a prodrug that requires metabolic conversion to CLO to exert its full insecticidal effect^58,59^. CLO exhibits strong agonistic activity at insect nAChRs and slightly weaker toxicity than TMX (LC50 of 30.25 µM for TMX and 39.37 µM for CLO, Supplementary Fig. 4)^58,60^.

**Fig. 4:**
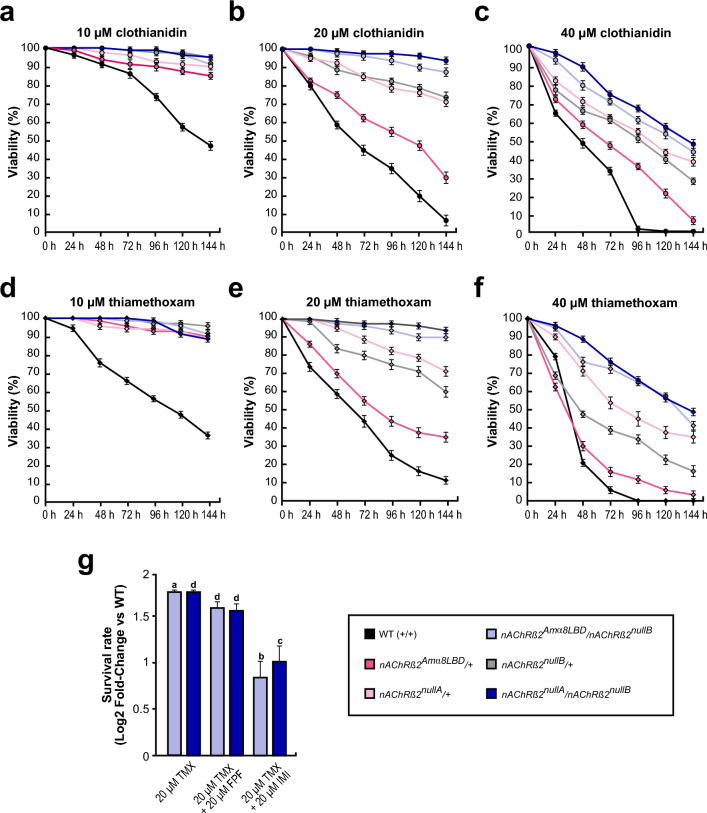
The α8 ligand binding domain does not sensitise flies to neonicotinoids. (**a**–**f**) Viability of flies exposed to 10 µM (a, d), 20 µM (b, e) and 40 µM (c, f) clothianidin (a-c) or thiamethoxam (d-f) measured every 24 h for 6 days is shown as mean with standard error from four biologically independent groups starting with 20 flies. (**g**) Survival rate of *nAChRβ2*^*Amα8LBD*^/*nAChRβ2*^*nullA*^ and *nAChRβ2*^*nullA*^/*nAChRβ2*^*nullB*^ mutants compared to WT flies subjected to different insecticides combinations. The log2 fold-change shown as mean with standard error from four biologically independent groups starting with 20 flies represents the viability of flies after six days of treatment with TMX (*p* = 0.0429 for *nAChRβ2*^*Amα8LBD*^/*nAChRβ2*^*nullB*^ and* p* = 4.2*10^–5^ for *nAChRβ2*^*nullA*^/*nAChRβ2*^*nullB*^) or TMX mixed with flupyradifurone (FPF; *p* = 2.8*10^–5^ for *nAChRβ2*^*Amα8LBD*^/*nAChRβ2*^*nullB*^ and *p* = 1.3*10^–5^ for *nAChRβ2*^*nullA*^/*nAChRβ2*^*nullB*^) or imidacloprid (IMI; *p* = 0.0121 for *nAChRβ2*^*Amα8LBD*^/*nAChRβ2*^*nullB*^ and *p* = 0.0021 for *nAChRβ2*^*nullA*^/*nAChRβ2*^*nullB*^) in equimolar ratios. Statistically significant differences are indicated with letters (a = *p ≤ 0.5, b = **p ≤ 0.01, c = ***p ≤ 0.001, d = ****p ≤ 0.0001).

Next, we measured toxicity over time at different TMX and CLO concentrations. At 10 μM, wild type flies showed 43.75 and 47.5% survival at 144 h for CLO and TMX, respectively. In contrast, *nAChRβ2*^*Amα8LBD*^*/* + , *nAChRβ2*^*nullA*^*/* + , *nAChRβ2*^*nullB*^*/* + , *nAChRβ2*^*nullA*^*/nAChRβ2*^*nullB*^, and *nAChRβ2*^*Amα8LBD*^/*nAChRβ2*^*nullB*^ flies remained viable (Fig. 4a,d).

At 20 μM TMX and CLO, survival declined progressively across all genotypes. *nAChRβ2*^*nullA*^/*nAChRβ2*^*nullB*^ mutants showed the highest viability, followed by *nAChRβ2*^*Amα8LBD*^/*nAChRβ2*^*nullB*^ flies, which retained approximately 90% viability by day 6. In contrast, *nAChRβ2*^*nullA*^*/* + (71.25% and 72.15%), *nAChRβ2*^*nullB*^*/* + (60 and 73.75%), *nAChRβ2*^*Amα8LBD*^*/* + (35% and 30%), and WT (11.25% and 7.75%) exhibited markedly lower survival (Fig. 4b,e).

At 40 μM TMX and CLO, all genotypes showed less than 50% viability by 144 h. *nAChRβ2*^*nullA*^/*nAChRβ2*^*nullB*^ mutants retained 48.75% (TMX) and 47.5% (CLO) survival, followed by *nAChRβ2*^*Amα8LBD*^/*nAChRβ2*^*nullB*^ (41.25% and 43.75%), *nAChRβ2*^*nullA*^*/* + (35 and 37.5%), *nAChRβ2*^*nullB*^*/* + (16.25% and 26.25%), *nAChRβ2*^*Amα8LBD*^*/* + (3.33% and 7.5%), while WT flies exhibited complete lethality by day 4 (TMX) and 5 (CLO) (Fig. 4c,f). Interestingly, apart from the trans-heterozygous *β*^*nullA*^/*β*^*nullB*^ flies, all genotypes experienced a sharp drop in viability between the 1st and 2nd day of treatment with TMX, particularly *nAChRβ2*^*Amα8LBD*^*/* + dropping from 62.5 to 30% and WT failing from 79 to 20%.

TMX and CLO primarily target nAChR subtypes containing Dβ1, and to a lesser extent, Dα1 and Dα3 subunits^50,61,62^. In contrast, imidacloprid (IMI) acts on receptor subtypes comprising Dα1, Dα2, Dβ1 and Dβ2 subunits, with lower affinity for those containing Dα4 and Dα7 subunits^50,61^. Flupyradifurone (FPF), a systemic butanolide compound chemically distinct from neonicotinoids representing a novel alternative to these compounds in pest management^63^. FPF acts on receptor subtypes having Dβ1 subunits and, to a lesser degree, to receptor subtypes having Dα3^50^ and exerts broad-spectrum activity and demonstrated toxicity to non-target pollinators, including bees^64,65^.

TMX, IMI and FPF act as nAChR agonists, bind to the same site and cause prolonged activation, they vary in the binding dynamics, receptor subtype preferences, and potential to develop resistance^66^. Given the different mechanisms of toxicity of TMX compared to IMI and FPF, we tested whether IMI and FPF would potentiate toxicity of TMX in combination. We exposed flies to a mixture of TMX (20 μM) with either FPF (20 μM) or IMI (20 μM). In nature, pollinators and insects are frequently exposed to multiple insecticides rather than a single compound, making it crucial to understand potential interactions and cross-resistance effects^67,68^. The TMX concentration was chosen based on prior survival analyses, where double mutant flies retained > 90% viability (Fig. 4e), while WT flies showed significant susceptibility. We tested chimeric *nAChRβ2*^*Amα8LBD*^/*nAChRβ2*^*nullB*^ flies and *nAChRβ2*^*nullA*^/*nAChRβ2*^*nullB*^ mutants to determine whether altered receptor composition influences responses to dual insecticide exposure. Flies were treated for six days, and viability on day six was analysed as a log-fold change relative to WT control and TMX-only treatment (Fig. 4g). Notably, exposure to TMX in combination with either FPF (*p* = 2.8*10^–5^ for *nAChRβ2*^*Amα8LBD*^/*nAChRβ2*^*nullB*^ and *p* = 1.3*10^–5^ for *nAChRβ2*^*nullA*^/*nAChRβ2*^*nullB*^) or IMI (*p* = 0.0121 for *nAChRβ2*^*Amα8LBD*^/*nAChRβ2*^*nullB*^ and *p* = 0.0021 for *nAChRβ2*^*nullA*^/*nAChRβ2*^*nullB*^) resulted in a slight increase in survival rates. However, the combined treatment did not greatly alter overall insecticide susceptibility. As expected, *nAChRβ2*^*Amα8LBD*^/*nAChRβ2*^*nullB*^ flies and *nAChRβ2*^*nullA*^/*nAChRβ2*^*nullB*^ mutants flies exhibited significantly higher resistance compared to WT across all conditions, including TMX-only treatment (*p* = 0.0429 for *nAChRβ2*^*Amα8LBD*^/*nAChRβ2*^*nullB*^ and *p* = 4.2*10^–5^ for *nAChRβ2*^*nullA*^/*nAChRβ2*^*nullB*^).

Taken together, our findings demonstrate that the β2^null^ (*nAChRβ2*^*nullA*^/*nAChRβ2*^*nullB*^) mutants, as well as the LBD swap (*nAChRβ2*^*Amα8LBD*^*/nAChRβ2*^*nullB*^) flies exhibit higher survival rates upon exposure to TMX, reinforcing the role of Dβ2 in neonicotinoid susceptibility. Further, exposure to combined neonicotinoid and butanolide treatments did not significantly alter overall lethality compared to TMX treatment on its own, indicating that sub-lethal doses of these nAChR-targeting insecticides do not act synergistically in this context. The observed resistance of chimeric and β2^null^ mutants further highlights the functional divergence of Amelα8 in modifying nAChR-mediated insecticide susceptibility.

## Discussion

The agricultural industry relies on pesticides to protect crops and maintain a stable food supply for a growing population. Among these, neonicotinoids are the most widely used insecticides due to their high efficacy and systemic action, reducing the need for repeated applications. However, their persistence in plant tissues, including nectar and pollen, poses a serious threat to pollinators. Despite well-documented sublethal and lethal effects on bees and other beneficial insects, neonicotinoids remain the dominant insecticides worldwide^69^, highlighting the urgent need to mitigate their ecological impact.

Neonicotinoid toxicity varies widely across species. *Drosophila melanogaster*, for instance, is considerably more resistant than honey bees, despite homologous nAChR subunits^61^, raising the question of whether sequence identity between subunits is sufficient to explain these differences. To understand whether structural conservation confers insecticide resistance, we directly tested the contribution of the LBD to neonicotinoid sensitivity. Thus, we used CRISPR-Cas9 to generate a chimeric *Drosophila* mutant expressing a β2 subunit in which the LBD was replaced with that of honey bee Amelα8. Contrary to expectations, the resulting chimeric receptor did not sensitise fly viability to neonicotinoid exposure. However, the LBD swap mutant displayed motor function deficits, indicating that the chimeric receptor forms functional channels that affect motor behaviour. These findings demonstrate that sequence identity alone does not predict pharmacological response and is insufficient to recapitulate the function of homologous subunit.

Nevertheless, even minor structural changes in receptor subunits can profoundly impact pharmacological properties^36,38,49,50^. Specific amino acid substitutions in nAChR subunits are known to alter insecticide susceptibility as found for the R81T substitution in the aphid β1 subunit. Also *Drosophila* gene knockouts of individual subunits exhibit an increase in resistance to neonicotinoids and other insecticides^48–50,70^. More broadly, species-specific differences in sensitivity likely result from variation in receptor composition, metabolism, or detoxification pathways^29,31^, though the relative contribution of each remains unclear.

Functional diversity in nAChR subunit expression adds further complexity. In honey bees, α2, α8 and β1 are expressed in Kenyon cells, while α7 is restricted to antennal lobe neurons^71^. In *Drosophila*, α1 and α6 contribute to dendrite morphogenesis and synaptic transmission in larval visual circuits^72^. Developmental reprogramming of nAChR expression, such as reduced Dα3 in larvae and stage-specific expression profile shifts of subunits, may support behavioural role-dependent specialisations of different subunits, including reproduction^62,73^. Furthermore, plasticity is not restricted to development. Subchronic exposure to sub-lethal doses of imidacloprid in *Periplaneta americana* induces a significant decrease in α2 mRNA expression, and reduces sensitivity to the insecticide^74^, highlighting potential adaptive changes in nAChR subunit composition even in fully developed insects.

Furthermore, high *Dβ2* expression in CNS-specific neuronal populations, including Kenyon cells, suggests its role in higher-order functions such as learning and memory^54^. Similarly, *Amelα8* is enriched in honey bee mushroom bodies^43,75,76^, suggesting a conserved role across insect taxa, despite species-specific specialisations. Notably, neonicotinoid exposure disrupts cell activity in mushroom body and impairs learning and memory in insects, including *Drosophila* and honey bees^24–26,77^, further highlighting the critical role of nAChRs in cognitive functions.

Pharmacological and genetic studies have identified α1, α2, β1, and β2 as the primary mediators of neonicotinoid toxicity^40,61,78^, however, identifying the molecular determinants of binding and downstream signalling underlying species-specific effects require further investigation of receptor stoichiometry and subunit interfaces. Our finding that the α8β2 chimeric mutant did not exhibit increased sensitivity indicates that homology at the LBD level is insufficient to account for species-specific pharmacological differences. This challenges the assumption that sequence similarity alone is predictive of toxicological response. Instead, interactions at subunit interfaces, receptor assembly dynamics, and accessory proteins may be critical determinants of response. Clarifying these features is essential for understanding what governs neonicotinoid binding and efficacy across taxa.

In conclusion, our study establishes a framework for investigating molecular determinants of neonicotinoid sensitivity of insects and vertebrates nAChR using a cross-species chimeric nAChR *Drosophila* model. This model can contribute to the design of next generation of insecticides for enhanced species-specific responses with implications for pollinator conservation and pesticide safety for vertebrates including humans.

## Materials and methods

### Fly stocks, genetics, immunostaining of tissues and imaging

*D. melanogaster* CantonS and w^1118^ were used as the wild type control. *nAChRß2*^*attP*^ (BDSC 84545) and *nAChRβ2*^*[2A-GAL4]*^ (BDSC 84666) stocks were described previously^57^ and were together with the chromosomal deficiency (BDSC 24996) from Bloomington. Fly crosses were maintained at 25 °C in plastic vials containing 10 ml of a standard cornmeal/yeast-rich medium (1% agar, 2% yeast, 7% dextrose, 8% cornmeal w/v and 2% Nipagin from a 10% solution in ethanol) with a 12:12 h light–dark cycle.

Third instar wandering larvae and adult brains from the progeny of *UAS-Histone2B::YFP/* + ;*nAChRβ2*^*[2A-GAL4]*^*/* + and *UAS-mCD8::GFP/* + ;*nAChRβ2*^*[2A-GAL4]*^*/* + were dissected in phosphate buffered saline (PBS) and fixed in 4% paraformaldehyde in PBT (PBS with 0.1% TritonTM X-100 (Sigma-Aldrich, T8787)) for 30 min, followed by washes in PBT 3 × 15 min. Samples were incubated overnight at 4 °C with primary mouse anti-*elav* antibodies (MAb 7D, 1:20)^79^, followed by secondary antibodies conjugated with Alexa Fluor 546 again overnight at 4 °C. Samples were counterstained with DAPI (1:1000), mounted in Vectashield (Vector Labs), scanned with Leica SP8, and processed using FIJI.

### Sequence analysis and single cell expression data visualisation

Amino acid sequences were aligned using ClustalW with Megalin (DNAstar) or with MAFFT^80^ and phylogenetic trees were generated in NGPhylogeny.fr^81–83^ and visualised using iTOL^84^.

Raw whole-genome sequencing reads were assessed for quality using FastQC, followed by adapter trimming and quality filtering with Trim Galore. High-quality reads were aligned to the *Drosophila melanogaster* (dm6) and *Apis mellifera* (Amel_HAv3.1) reference genomes using BBMap. Mapping quality and coverage were evaluated, and alignments were visualised using IGV (Integrative Genomics Viewer).

Single cell expression data was visualised as t-distributed stochastic neighbour embedding (tSNE) from the 10 × Stringent dataset in Scope and ASAP^85^.

### Generation of chimeric *nAChRα8β2* flies

Two single guide RNAs (sgRNAs) flanking the ligand binding domain *Dβ2* were designed using PlatinumCRISPr^86^ (Supplementary Fig. 3a,b) and cloned into *pUC-3GLA* using the following primers: nAChRα8 sgRNA left F1 (AAGATATCCGGGTGAACTTCGCTTATTGGAGCTAGGAAAGGTTTTAGAGCTAGAAATAGC) and nAChRα8 sgRNA right R1 (GCTATTTCTAGCTCTAAAACCACATGGCACAATCAAATTCGACGTTAAATTGAAAATAGG) as described previously^86^. Chimeric nAChR subunits were constructed by combining sequences of *nAChRα8* from *A. mellifera* and *nAChRβ2* from *D. melanogaster* in three steps (Supplementary Fig. 3c–e). Fragments used for generating the chimeric nAChR subunit were synthesised using PCR with primers Beeα8 F2 (GGGAGTGGTCATTGCCATCTCAACCCTATATAAATTTG) and Beeα8 R2 (GGAATGTAATACCCACGCAAGGAATTATTAAG), followed by Beeα8 F1 (GCCAGTGAATTCGAGCTCGGTACCGCAGCTAGCGAAGCAAATCCTGACACAAAGAGACTTTATGATGAC) and Beeα8 R1 (TTTACGCAGACTATCTTTCTAGGGTTAACCGTATAGAATAATGTTTTTCTTCGCATTG) for Amelα8 ligand binding domain, Dmel nAChRβ2 left F1 (CGGCCAGTGAATTCGAGCTCGGTACCGATCCTTTTAGATAAAACATTTAGGAGCTATC) and nAChRsgmut left R1 (CTCTTTGTGTCAGGATTTGCTTCGAAACTCACTGGAGCCGTGAGAGGAGGGGTAATTCAGGGGAAAAACAGAGAAAAATGCC) for Dβ2 left homology arm, and nAChRsgmut right F1 (ATTTTACGCATGATTATCTTTAACGTACGTCACAATATGATTATCTTTCTAGGGTTAATCTGATTGTGCCCTGCGTAGCTTTAACATTCC) and Dmelα8 right R1 (GCATGCCTGCAGGTCGACTCTAGAGGATCCTGATAGTTGCTGCTGCGAATGCGGAGCTG) for Dβ2 right homology arm, ensuring sgRNA sites in both homology arms are mutated. Mutations were introduced based on conservation of the region between closely related *Drosophila* species analysed using the UCSC genome browser (https://genome.ucsc.edu) as described previously^87^. Fragments were then cloned stepwise into the *pUC19 pBac w* + [accession number: PV267745] containing *white* + marker, disrupting the LBD, therefore resulting in *nAChRβ2*^*null*^ flies. The selection marker was flanked with inverted terminal repeats inserted at a TTAA PiggyBac motifs for the later scarless excision by PiggyBac transposase and restoring the open reading frame resulting in a fly stock expressing the chimeric *nAChRβ2/α8* subunit (*nAChRβ2*^*Amα8LBD*^). The *pUC19 pBac w* + *Amelα8/Dβ2* plasmid was treated with the nicking endonuclease *Nb.Bts*I (NEB, R0707S) prior to injection. Transgenic lines were generated by injection into a GFP nosCas9 carrying flies (kindly provided by FlyORF). A total of three injections were done with supercoiled plasmids (0.4 µg/µl repair template and 0.1 µg/µl sgRNA plasmid) yielding 186, 176 and 141 survivors with a fertility rate of 67, 62 and 55%, but no transformants were found. From one injection with a nicked repair template, we obtained 135 survivors with a fertility rate of 65% and we got two G0 flies with transformants. Positive transformants were identified by red eye colour. Flies were validated by PCR, Sanger sequencing, and subsequently by whole genome sequencing.

For *Drosophila* whole genome sequencing, large fragment DNA was extracted with the Quick-DNA Tissue/Insect Miniprep Kit (Zymogene) according to the manufacturers’ instructions but replacing the BashingBead lysis step with cracking flies in liquid nitrogen followed by gentle homogenization with a pestle in the provided BashingBead Buffer. Illumina sequencing was done by Novogene and the sequence submitted to GEO [PRJNA1233772].

### Locomotive assessment

Negative geotaxis experiments were done as previously described^88^. Briefly, two to five day-old flies of both sexes were collected with CO_2_ anaesthesia and grouped in sets of 20. Flies were allowed to recover for a day and then placed in two inverted fly vials (19 cm). Flies were tapped to the bottom, and their climbing behaviour was recorded on video. Every 5 s for a total of 30 s, the distance climbed by each fly was measured and recorded. The data was collected for four biological replicates.

For flying ability assessment, two to five day-old flies of both sexes were collected with CO_2_ anaesthesia and grouped in sets of 10. Flies were allowed to recover for a day before the flying test. Then, flies were tapped to a flat surface, and their ability to fly was recorded on video. Flies were observed over a 30 s period. At the end of 30 s, the number of flies that successfully flew away was recorded. The data was collected for four biological replicates.

### Insecticide toxicity assay

Two to five day-old flies of both sexes, grouped in sets of 20, were exposed to various concentrations of clothianidin (Sigma-Aldrich, T21153), thiamethoxam (Sigma-Aldrich, 37,924), or a combination of 20 µM of thiamethoxam with 20 µM imidacloprid (Thermo Fisher, 466,752,500) or 20 µM flupyradifurone (MCE, HY-145295). The insecticides were dissolved in 0.01% acetone and added on top of the standard fly food, and flies were added to the vials after 24 h. A full dose–response survival assay was performed for the wild type genotype only. Flies were exposed to thiamethoxam and clothianidin concentrations ranging from 2.5 to 80 µM (Supplementary Fig. 4). A generalised linear model (GLM) was used to estimate the relationship between dose and mortality, yielding an LC50 of 30.25 µM for thiamethoxam and 39.37 µM for clothianidin (see Statistical analysis section). Mutant genotypes were tested at fixed concentrations based on the wild type response and ranged from 10 to 40 µM for all genotypes. Flies were exposed for 6 days, and survival was assessed every 24 h.

### Statistical analysis

Statistical analyses were carried out using GraphPad Prism 9 and R (v4.4.1). One-way ANOVA followed by Tukey’s post hoc test was used for behavioural assays. For insecticide toxicity, GLM with a binomial error distribution and logit link were fitted to mortality data (cbind(dead, alive) ~ conc) using the glm() function in R. Model fit was assessed using residual deviance and AIC. Significance of the concentration effect was determined using Wald tests. To test for synergistic effects, survival under combined treatments (TMX + IMI or TMX + FPF) was compared to survival under TMX-only exposure using two-way ANOVA followed by Tukey’s test for pairwise comparisons. All data represent a minimum of three independent replicates conducted on separate days.

## Supplementary Information

Supplementary Information.

## Data Availability

The data generated or analysed during this study are included in the supplementary information files. Whole genome sequencing data have been deposited at GEO under the accession number PRJNA1233772. The sequence for the *pUC19 pBac w +*  has been deposited in GenBank under the accession number PV267745 and the plasmid is available from Addgene and the European plasmid repository.

## References

[1] Fenik, J., Tankiewicz, M. & Biziuk, M. Properties and determination of pesticides in fruits and vegetables. *TrAC Trends Anal. Chem.* **30**, 814–826 (2011).

[2] Elbert, D. L., Mawuenyega, K. G., Scott, E. A., Wildsmith, K. R. & Bateman, R. J. Stable isotope labeling tandem mass spectrometry (SILT): Integration with peptide identification and extension to data-dependent scans. *J. Proteome Res.* **7**, 4546–4556 (2008). 18774841 10.1021/pr800386u PMC2707264

[3] Jeschke, P., Nauen, R., Schindler, M. & Elbert, A. Overview of the status and global strategy for neonicotinoids. *J. Agric. Food Chem.* **59**, 2897–2908 (2011). 20565065 10.1021/jf101303g

[4] Li, P., Ann, J. & Akk, G. Activation and modulation of human α4β2 nicotinic acetylcholine receptors by the neonicotinoids clothianidin and imidacloprid. *J. Neurosci. Res.* **89**, 1295–1301 (2011). 21538459 10.1002/jnr.22644 PMC3668458

[5] Gibbons, D., Morrissey, C. & Mineau, P. A review of the direct and indirect effects of neonicotinoids and fipronil on vertebrate wildlife. *Environ. Sci. Pollut. Res.* **22**, 103–118 (2015). 10.1007/s11356-014-3180-5 PMC4284370 24938819

[6] Burke, A. P. et al. Mammalian susceptibility to a neonicotinoid insecticide after fetal and early postnatal exposure. *Sci. Rep.* **8**, 16639 (2018). 30413779 10.1038/s41598-018-35129-5 PMC6226530

[7] Casida, J. E. Neonicotinoids and other insect nicotinic receptor competitive modulators: Progress and prospects. *Annu. Rev. Entomol.* **63**, 125–144 (2018). 29324040 10.1146/annurev-ento-020117-043042

[8] Costas-Ferreira, C. & Faro, L. R. F. Neurotoxic effects of neonicotinoids on mammals: What is there beyond the activation of nicotinic acetylcholine receptors?—a systematic review. *Int. J. Mol. Sci.* **22**, 8413 (2021). 34445117 10.3390/ijms22168413 PMC8395098

[9] Henry, M. et al. A common pesticide decreases foraging success and survival in honey bees. *Science* **336**, 348–350 (2012). 22461498 10.1126/science.1215039

[10] Pisa, L. W. et al. Effects of neonicotinoids and fipronil on non-target invertebrates. *Environ. Sci. Pollut. Res.* **22**, 68–102 (2015). 10.1007/s11356-014-3471-x PMC4284392 25223353

[11] Woodcock, B. A. et al. Impacts of neonicotinoid use on long-term population changes in wild bees in England. *Nat. Commun.* **7**, 12459 (2016). 27529661 10.1038/ncomms12459 PMC4990702

[12] Crall, J. D. et al. Neonicotinoid exposure disrupts bumblebee nest behavior, social networks, and thermoregulation. *Science* **362**, 683–686 (2018). 30409882 10.1126/science.aat1598

[13] Gill, R. J., Ramos-Rodriguez, O. & Raine, N. E. Combined pesticide exposure severely affects individual- and colony-level traits in bees. *Nature* **491**, 105–108 (2012). 23086150 10.1038/nature11585 PMC3495159

[14] Muth, F. & Leonard, A. S. A neonicotinoid pesticide impairs foraging, but not learning, in free-flying bumblebees. *Sci. Rep.* **9**, 4764 (2019). 30886154 10.1038/s41598-019-39701-5 PMC6423345

[15] Rundlöf, M. et al. Seed coating with a neonicotinoid insecticide negatively affects wild bees. *Nature* **521**, 77–80 (2015). 25901681 10.1038/nature14420

[16] Tasman, K., Rands, S. A. & Hodge, J. J. L. Using radio frequency identification and locomotor activity monitoring to assess sleep, locomotor, and foraging rhythmicity in bumblebees. *STAR Protoc.* **2**, 100598 (2021). 34169292 10.1016/j.xpro.2021.100598 PMC8209741

[17] Tasman, K., Rands, S. A. & Hodge, J. J. L. The neonicotinoid insecticide imidacloprid disrupts bumblebee foraging rhythms and sleep. *iScience* **23**, 101827 (2020). 33305183 10.1016/j.isci.2020.101827 PMC7710657

[18] Whitehorn, P. R., O’Connor, S., Wackers, F. L. & Goulson, D. Neonicotinoid pesticide reduces bumble bee colony growth and queen production. *Science* **336**, 351–352 (2012). 22461500 10.1126/science.1215025

[19] Forfert, N. et al. Neonicotinoid pesticides can reduce honeybee colony genetic diversity. *PLoS ONE* **12**, e0186109 (2017). 29059234 10.1371/journal.pone.0186109 PMC5653293

[20] Annoscia, D. et al. Neonicotinoid clothianidin reduces honey bee immune response and contributes to Varroa mite proliferation. *Nat. Commun.* **11**, 5887 (2020). 33208729 10.1038/s41467-020-19715-8 PMC7675992

[21] Decio, P. et al. Thiamethoxam exposure deregulates short ORF gene expression in the honey bee and compromises immune response to bacteria. *Sci. Rep.* **11**, 1489 (2021). 33452318 10.1038/s41598-020-80620-7 PMC7811001

[22] Di Prisco, G. et al. Neonicotinoid clothianidin adversely affects insect immunity and promotes replication of a viral pathogen in honey bees. *Proc. Natl. Acad. Sci.* **110**, 18466–18471 (2013). 24145453 10.1073/pnas.1314923110 PMC3831983

[23] Oliveira, R. A., Roat, T. C., Carvalho, S. M. & Malaspina, O. Side-effects of thiamethoxam on the brain andmidgut of the africanized honeybee *Apis mellifera* (Hymenopptera: Apidae). *Environ. Toxicol.* **29**, 1122–1133 (2014). 23339138 10.1002/tox.21842

[24] Palmer, M. J. et al. Cholinergic pesticides cause mushroom body neuronal inactivation in honeybees. *Nat. Commun.* **4**, 1634 (2013). 23535655 10.1038/ncomms2648 PMC3621900

[25] Smith, D. B. et al. Insecticide exposure during brood or early-adult development reduces brain growth and impairs adult learning in bumblebees. *Proc. R. Soc. B Biol. Sci.* **287**, 20192442 (2020). 10.1098/rspb.2019.2442 PMC7126076 32126960

[26] Tasman, K., Hidalgo, S., Zhu, B., Rands, S. A. & Hodge, J. J. L. Neonicotinoids disrupt memory, circadian behaviour and sleep. *Sci. Rep.* **11**, 2061 (2021). 33479461 10.1038/s41598-021-81548-2 PMC7820356

[27] Tatarko, A. R., Leonard, A. S. & Mathew, D. A neonicotinoid pesticide alters Drosophila olfactory processing. *Sci. Rep.* **13**, 10606 (2023). 37391495 10.1038/s41598-023-37589-w PMC10313779

[28] Li, X., Liu, J. & Wang, X. Exploring the multilevel hazards of thiamethoxam using *Drosophila melanogaster*. *J. Hazard. Mater.* **384**, 121419 (2020). 31630861 10.1016/j.jhazmat.2019.121419

[29] Berenbaum, M. R. & Johnson, R. M. Xenobiotic detoxification pathways in honey bees. *Curr. Opin. Insect Sci.* **10**, 51–58 (2015). 29588014 10.1016/j.cois.2015.03.005

[30] Tasman, K., Rands, S. A. & Hodge, J. J. L. The power of drosophila melanogaster for modeling neonicotinoid effects on pollinators and identifying novel mechanisms. *Front. Physiol.* **12**, 659440 (2021). 33967830 10.3389/fphys.2021.659440 PMC8096932

[31] Corona, M. & Robinson, G. E. Genes of the antioxidant system of the honey bee: annotation and phylogeny. *Insect Mol. Biol.* **15**, 687–701 (2006). 17069640 10.1111/j.1365-2583.2006.00695.x PMC1847502

[32] Tomizawa, M. & Casida, J. E. Structure and diversity of insect nicotinic acetylcholine receptors. *Pest Manag. Sci.* **57**, 914–922 (2001). 11695184 10.1002/ps.349

[33] Role, L. W. & Berg, D. K. Nicotinic receptors in the development and modulation of CNS synapses. *Neuron* **16**, 1077–1085 (1996). 8663984 10.1016/s0896-6273(00)80134-8

[34] Wessler, I. & Kirkpatrick, C. J. Acetylcholine beyond neurons: the non-neuronal cholinergic system in humans. *Br. J. Pharmacol.* **154**, 1558–1571 (2008). 18500366 10.1038/bjp.2008.185 PMC2518461

[35] Tomizawa, M., Millar, N. S. & Casida, J. E. Pharmacological profiles of recombinant and native insect nicotinic acetylcholine receptors. *Insect Biochem. Mol. Biol.* **35**, 1347–1355 (2005). 16291090 10.1016/j.ibmb.2005.08.006

[36] Matsuda, K. et al. Neonicotinoids: insecticides acting on insect nicotinic acetylcholine receptors. *Trends Pharmacol. Sci.* **22**, 573–580 (2001). 11698101 10.1016/s0165-6147(00)01820-4

[37] Karlin, A. Emerging structure of the nicotinic acetylcholine receptors. *Nat. Rev. Neurosci.* **3**, 102–114 (2002). 11836518 10.1038/nrn731

[38] Ihara, M. et al. Crystal structures of *Lymnaea stagnalis* AChBP in complex with neonicotinoid insecticides imidacloprid and clothianidin. *Invert. Neurosci.* **8**, 71–81 (2008). 18338186 10.1007/s10158-008-0069-3 PMC2413115

[39] Andersen, N., Corradi, J., Sine, S. M. & Bouzat, C. Stoichiometry for activation of neuronal α7 nicotinic receptors. *Proc. Natl. Acad. Sci.* **110**, 20819–20824 (2013). 24297903 10.1073/pnas.1315775110 PMC3870720

[40] Jones, A. K. & Sattelle, D. B. Diversity of insect nicotinic acetylcholine receptor subunits. In *Insect Nicotinic Acetylcholine Receptors* (ed. Thany, S. H.) (Springer, 2010). 10.1007/978-1-4419-6445-8_3 20737786

[41] Taillebois, E. & Thany, S. H. Characterization of nicotine acetylcholine receptor subunits in the cockroach *Periplaneta americana* mushroom bodies reveals a strong expression of beta1 subunit: involvement in nicotine-induced currents. *Arch. Insect Biochem. Physiol.* **93**, 40–54 (2016). 27357353 10.1002/arch.21340

[42] Millar, N. S. & Gotti, C. Diversity of vertebrate nicotinic acetylcholine receptors. *Neuropharmacology* **56**, 237–246 (2009). 18723036 10.1016/j.neuropharm.2008.07.041

[43] Dupuis, J., Louis, T., Gauthier, M. & Raymond, V. Insights from honeybee (*Apis mellifera*) and fly (*Drosophila melanogaster*) nicotinic acetylcholine receptors: From genes to behavioral functions. *Neurosci. Biobehav. Rev.* **36**, 1553–1564 (2012). 22525891 10.1016/j.neubiorev.2012.04.003

[44] Jones, A. K., Goven, D., Froger, J., Bantz, A. & Raymond, V. The cys-loop ligand-gated ion channel gene superfamilies of the cockroaches *Blattella germanica* and *Periplaneta americana*. *Pest Manag. Sci.* **77**, 3787–3799 (2021). 33347700 10.1002/ps.6245

[45] Noviello, C. M. et al. Structure and gating mechanism of the α7 nicotinic acetylcholine receptor. *Cell* **184**, 2121-2134.e13 (2021). 33735609 10.1016/j.cell.2021.02.049 PMC8135066

[46] Shimomura, M., Yokota, M., Matsuda, K., Sattelle, D. B. & Komai, K. Roles of loop C and the loop B-C interval of the nicotinic receptor α subunit in its selective interactions with imidacloprid in insects. *Neurosci. Lett.* **363**, 195–198 (2004). 15182942 10.1016/j.neulet.2003.12.115

[47] Shimada, S. et al. The mechanism of loop C-neonicotinoid interactions at insect nicotinic acetylcholine receptor α1 subunit predicts resistance emergence in pests. *Sci. Rep.* **10**, 7529 (2020). 32371996 10.1038/s41598-020-64258-z PMC7200709

[48] Bass, C. et al. Mutation of a nicotinic acetylcholine receptor β subunit is associated with resistance to neonicotinoid insecticides in the aphid *Myzus persicae*. *BMC Neurosci.* **12**, 51 (2011). 21627790 10.1186/1471-2202-12-51 PMC3121619

[49] Homem, R. A. et al. Evolutionary trade-offs of insecticide resistance — The fitness costs associated with target-site mutations in the nAChR of *Drosophila melanogaster*. *Mol. Ecol.* **29**, 2661–2675 (2020). 32510730 10.1111/mec.15503 PMC7496652

[50] Lu, W. et al. Nicotinic acetylcholine receptor modulator insecticides act on diverse receptor subtypes with distinct subunit compositions. *PLOS Genet.* **18**, e1009920 (2022). 35045067 10.1371/journal.pgen.1009920 PMC8803171

[51] Jumper, J. et al. Highly accurate protein structure prediction with AlphaFold. *Nature* **596**, 583–589 (2021). 34265844 10.1038/s41586-021-03819-2 PMC8371605

[52] Krause, S. A., Overend, G., Dow, J. A. T. & Leader, D. P. FlyAtlas 2 in 2022: enhancements to the *Drosophila melanogaster* expression atlas. *Nucleic Acids Res.* **50**, D1010–D1015 (2022). 34718735 10.1093/nar/gkab971 PMC8728208

[53] Li, H. et al. Fly cell atlas: A single-nucleus transcriptomic atlas of the adult fruit fly. *Science* **375**, eabk2432 (2022). 35239393 10.1126/science.abk2432 PMC8944923

[54] Barnstedt, O. et al. Memory-relevant mushroom body output synapses are cholinergic. *Neuron* **89**, 1237–1247 (2016). 26948892 10.1016/j.neuron.2016.02.015 PMC4819445

[55] Ustaoglu, P. et al. Dynamically expressed single ELAV/Hu orthologue elavl2 of bees is required for learning and memory. *Commun. Biol.* **4**, 1234 (2021). 34711922 10.1038/s42003-021-02763-1 PMC8553928

[56] Brunet Avalos, C., Maier, G. L., Bruggmann, R. & Sprecher, S. G. Single cell transcriptome atlas of the Drosophila larval brain. *Elife* **8**, e50354 (2019). 31746739 10.7554/eLife.50354 PMC6894929

[57] Deng, B. et al. Chemoconnectomics: Mapping chemical transmission in Drosophila. *Neuron* **101**, 876-893.e4 (2019). 30799021 10.1016/j.neuron.2019.01.045

[58] Nauen, R., Ebbinghaus-Kintscher, U., Salgado, V. L. & Kaussmann, M. Thiamethoxam is a neonicotinoid precursor converted to clothianidin in insects and plants. *Pestic. Biochem. Physiol.* **76**, 55–69 (2003).

[59] Brown, L. A., Ihara, M., Buckingham, S. D., Matsuda, K. & Sattelle, D. B. Neonicotinoid insecticides display partial and super agonist actions on native insect nicotinic acetylcholine receptors. *J. Neurochem.* **99**, 608–615 (2006). 16899070 10.1111/j.1471-4159.2006.04084.x

[60] Tian, F. et al. Comparison of the effectiveness of thiamethoxam and its main metabolite clothianidin after foliar spraying and root irrigation to control *Myzus persicae* on peach. *Sci. Rep.* **12**, 16883 (2022). 36207356 10.1038/s41598-022-20659-w PMC9546927

[61] Ihara, M. et al. Cofactor-enabled functional expression of fruit fly, honeybee, and bumblebee nicotinic receptors reveals picomolar neonicotinoid actions. *Proc. Natl. Acad. Sci.* **117**, 16283–16291 (2020). 32611810 10.1073/pnas.2003667117 PMC7368294

[62] Komori, Y. et al. Functional impact of subunit composition and compensation on *Drosophila melanogaster* nicotinic receptors–targets of neonicotinoids. *PLOS Genet.* **19**, e1010522 (2023). 36795653 10.1371/journal.pgen.1010522 PMC9934367

[63] Nauen, R. et al. Flupyradifurone: a brief profile of a new butenolide insecticide. *Pest Manag. Sci.* **71**, 850–862 (2015). 25351824 10.1002/ps.3932 PMC4657471

[64] Tosi, S. et al. Long-term field-realistic exposure to a next-generation pesticide, flupyradifurone, impairs honey bee behaviour and survival. *Commun. Biol.* **4**, 805 (2021). 34183763 10.1038/s42003-021-02336-2 PMC8238954

[65] Siviter, H. et al. A novel pesticide has lethal consequences for an important pollinator. *Sci. Total Environ.* **952**, 175935 (2024). 39218110 10.1016/j.scitotenv.2024.175935

[66] Crossthwaite, A. J. et al. The invertebrate pharmacology of insecticides acting at nicotinic acetylcholine receptors. *J. Pestic. Sci.* **42**, 67–83 (2017). 30363948 10.1584/jpestics.D17-019 PMC6183333

[67] Johnson, R. M., Dahlgren, L., Siegfried, B. D. & Ellis, M. D. Acaricide, fungicide and drug interactions in honey bees (*Apis mellifera*). *PLoS ONE* **8**, e54092 (2013). 23382869 10.1371/journal.pone.0054092 PMC3558502

[68] Decio, P. et al. Acute thiamethoxam toxicity in honeybees is not enhanced by common fungicide and herbicide and lacks stress-induced changes in mRNA splicing. *Sci. Rep.* **9**, 19196 (2019). 31844097 10.1038/s41598-019-55534-8 PMC6915785

[69] Sparks, T. C. et al. Insecticides, biologics and nematicides: Updates to IRAC’s mode of action classification - a tool for resistance management. *Pestic. Biochem. Physiol.* **167**, 104587 (2020). 32527435 10.1016/j.pestbp.2020.104587

[70] Shi, X. G., Zhu, Y. K., Xia, X. M., Kang, Q. & Wang, K. Y. The mutation in nicotinic acetylcholine receptor β1 subunit may confer resistance to imidacloprid in *Aphis gossypii* (glover). *J. Food Agric. Environ.* **10**, 1227–1230 (2012).

[71] Cens, T. et al. Molecular targets of neurotoxic insecticides in *Apis mellifera*. *Eur. J. Org. Chem.* **2022**, e202101531 (2022).

[72] Rosenthal, J. S. et al. Temporal regulation of nicotinic acetylcholine receptor subunits supports central cholinergic synapse development in *Drosophila*. *Proc. Natl. Acad. Sci.* **118**, e2004685118 (2021). 34074746 10.1073/pnas.2004685118 PMC8202021

[73] Yoshinari, Y. et al. Neuronal octopamine signaling regulates mating-induced germline stem cell increase in female Drosophila melanogaster. *Elife* **9**, e57101 (2020). 33077027 10.7554/eLife.57101 PMC7591258

[74] Bantz, A., Goven, D., Siegwart, M., Maugin, S. & Raymond, V. Exposure to a sublethal dose of imidacloprid induces cellular and physiological changes in *Periplaneta americana*: Involvement of α2 nicotinic acetylcholine subunit in imidacloprid sensitivity. *Pestic. Biochem. Physiol.* **181**, 105014 (2022). 35082037 10.1016/j.pestbp.2021.105014

[75] Thany, S. H., Crozatier, M., Raymond-Delpech, V., Gauthier, M. & Lenaers, G. Apisα2, Apisα7-1 and Apisα7-2: three new neuronal nicotinic acetylcholine receptor α-subunits in the honeybee brain. *Gene* **344**, 125–132 (2005). 15656979 10.1016/j.gene.2004.09.010

[76] Thany, S. H., Lenaers, G., Crozatier, M., Armengaud, C. & Gauthier, M. Identification and localization of the nicotinic acetylcholine receptor alpha3 mRNA in the brain of the honeybee, *Apis mellifera*. *Insect Mol. Biol.* **12**, 255–262 (2003). 12752659 10.1046/j.1365-2583.2003.00409.x

[77] Ferreira, L. C. et al. Disruption of exploratory behavior and olfactory memory in cockroaches exposed to sublethal doses of the neonicotinoid Thiamethoxam. *Pestic. Biochem. Physiol.* **205**, 106167 (2024). 39477619 10.1016/j.pestbp.2024.106167

[78] Perry, T. et al. Role of nicotinic acetylcholine receptor subunits in the mode of action of neonicotinoid, sulfoximine and spinosyn insecticides in Drosophila melanogaster. *Insect Biochem. Mol. Biol.* **131**, 103547 (2021). 33548485 10.1016/j.ibmb.2021.103547

[79] Haussmann, I. U., Li, M. & Soller, M. ELAV-mediated 3′-end processing of *ewg* transcripts is evolutionarily conserved despite sequence degeneration of the ELAV-binding site. *Genetics* **189**, 97–107 (2011). 21705751 10.1534/genetics.111.131383 PMC3176107

[80] Katoh, K. & Standley, D. M. MAFFT multiple sequence alignment software version 7: Improvements in performance and usability. *Mol. Biol. Evol.* **30**, 772–780 (2013). 23329690 10.1093/molbev/mst010 PMC3603318

[81] Criscuolo, A. & Gribaldo, S. BMGE (Block Mapping and Gathering with Entropy): a new software for selection of phylogenetic informative regions from multiple sequence alignments. *BMC Evol. Biol.* **10**, 210 (2010). 20626897 10.1186/1471-2148-10-210 PMC3017758

[82] Guindon, S. et al. New algorithms and methods to estimate maximum-likelihood phylogenies: Assessing the performance of PhyML 3.0. *Syst. Biol.* **59**, 307–321 (2010). 20525638 10.1093/sysbio/syq010

[83] Lemoine, F. et al. NGPhylogeny.fr: new generation phylogenetic services for non-specialists. *Nucleic Acids Res.* **47**, W260–W265 (2019). 31028399 10.1093/nar/gkz303 PMC6602494

[84] Letunic, I. & Bork, P. Interactive Tree of Life (iTOL) v6: recent updates to the phylogenetic tree display and annotation tool. *Nucleic Acids Res.* **52**, W78–W82 (2024). 38613393 10.1093/nar/gkae268 PMC11223838

[85] David, F. P. A., Litovchenko, M., Deplancke, B. & Gardeux, V. ASAP 2020 update: an open, scalable and interactive web-based portal for (single-cell) omics analyses. *Nucleic Acids Res.* **48**, W403–W414 (2020). 32449934 10.1093/nar/gkaa412 PMC7319583

[86] Haussmann, I. U. et al. Structure-optimized sgRNA selection with PlatinumCRISPr for efficient Cas9 generation of knockouts. *Genome Res.* **34**, 2279–2292 (2024). 39626969 10.1101/gr.279479.124 PMC11694751

[87] McQuarrie, D. W. J. & Soller, M. Phylogenomic instructed target analysis reveals ELAV complex binding to multiple optimally spaced U-rich motifs. *Nucleic Acids Res.* **52**, 12712–12726 (2024). 39319593 10.1093/nar/gkae826 PMC11551757

[88] Haussmann, I. U. et al. CMTr cap-adjacent 2′-O-ribose mRNA methyltransferases are required for reward learning and mRNA localization to synapses. *Nat. Commun.* **13**, 1209 (2022). 35260552 10.1038/s41467-022-28549-5 PMC8904806

