# Supplementary material for: Assessing species-specific neonicotinoid toxicity using cross-species chimeric nicotinic acetylcholine receptors in a Drosophila model
Source: Sci Rep. 2025 Oct 16;15:36252. doi: 10.1038/s41598-025-20109-3 (PMC12533226; doi:10.1038/s41598-025-20109-3)
Supplement: Supplementary file 1 — Supplementary Information. [file 41598_2025_20109_MOESM1_ESM.pdf]

Supplementary Materials

for

Assessing species-specific neonicotinoid toxicity using cross-species chimeric nicotinic acetylcholine receptors in a *Drosophila* model

ANNA LASSOTA[1], JAMES J.L. HODGE[2] AND MATTHIAS SOLLER[1,3*]

Supplementary Figure 1

a **nAChRα8/ß2**

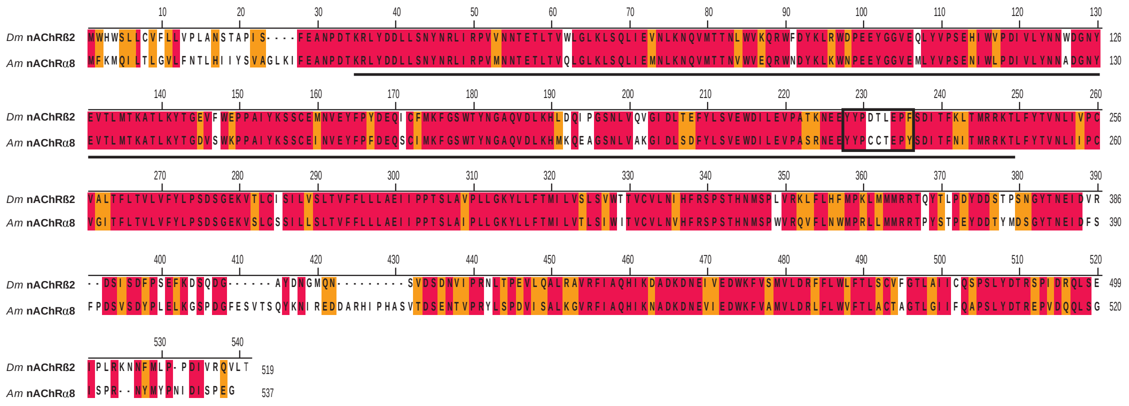

Supplementary Figure 1: Amino acid alignment of *Drosophila* Dβ2 and honey bee Amelα8

Amino acid alignment of *D. melanogaster* Dβ2 and honey bee Amelα8 subunits. Identical amino acids are marked in red, complementary amino acid substitutions are labelled in orange accordingly to the BLOSUM-62 substitution matrix which is a quantitative approach for assessing whether an amino acid substitution is conservative or nonconservative. Non-complementary amino acid substitutions are unmarked. The ligand binding domain is underlined, and the loop C is marked with a black box.

Supplementary Figure 2

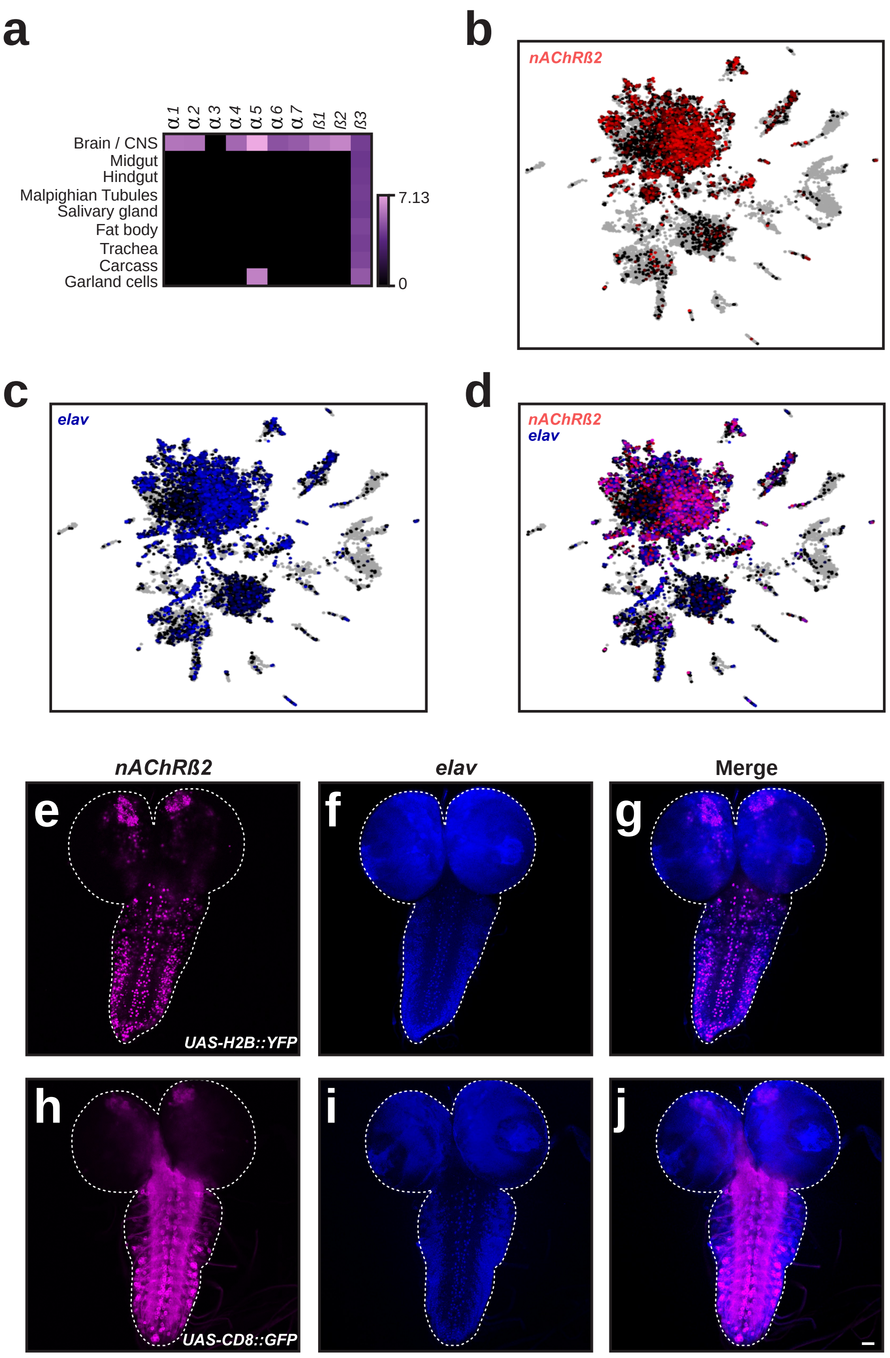

Supplementary Figure 2: *Dβ2* is highly expressed in larval *Drosophila* CNS but not in every cell

(a) Expression of all *Drosophila nAChR* subunits across various larval tissues obtained from FlyAtlas 2. Receptor subunits are indicated on top and tissues on the left. The colour scale bar indicates transcript abundance in fragments per kilobase of transcripts per million mapped reads (FPKM).

(b-d) Single-cell RNA-seq visualisation maps showing qualitative expression of *nAChRβ2* (red, b) on top of all cells (grey, b), the panneuronal marker *elav* (blue, c) or both (d) on top of all cells (grey) in the larval central nervous system from ASAP.

(e-j) Visual representation of *nAChRβ2* expression in larval *Drosophila* central nervous system using *UAS* reporter constructs driven by the endogenous promoter. Nuclear-localised (e-g) and membrane-bound (h-j) reporters highlight *Dβ2* spatial expression patterns. *Dβ2* expression (e, h) overlaps with the neuronal marker *elav* (f, i), as shown on merged pictures (g, j), confirming *Dβ2* expression in neuronal populations. Scale bar is 20 μm.

Supplementary Figure 3

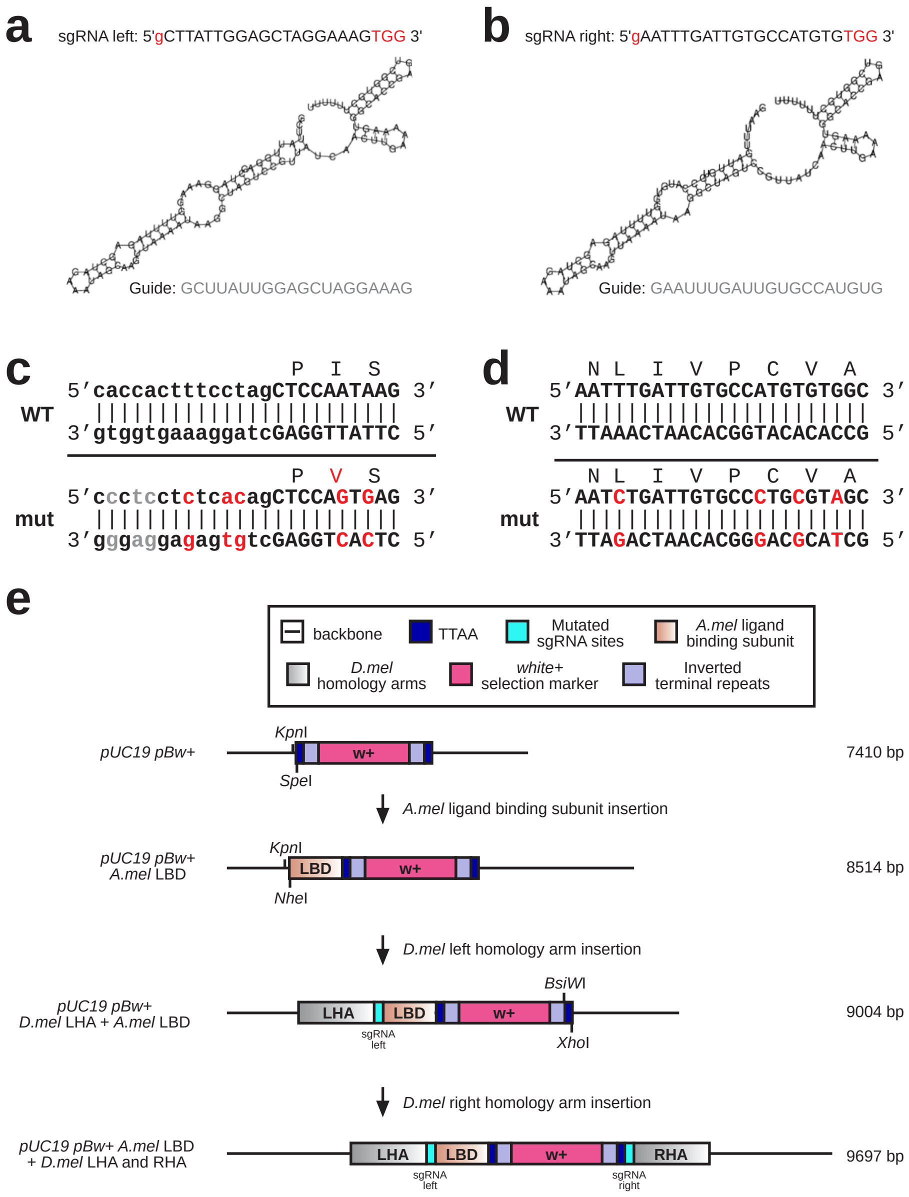

α

Supplementary Figure 3: Chimeric *nAChRβ2/α8* construct cloning strategy and sgRNA design

(a, b) Sequences and predicted secondary structures of single guide RNAs (sgRNAs) targeting regions upstream (“left”, a) and downstream (“right”, b) of the LBD. PAM sequence and additional G for enhanced base-pairing are highlighted in red.

(c, d) Endogenous (top) and mutated (bottom) sgRNAs sequences with open reading frames in letters are shown with nucleotide mutations indicated in red. The left sgRNA is on the reverse strand. Lower- and upper-case letters indicate intronic and exonic sequences, respectively. An I22V substitution was introduced to match the corresponding residue in the *A. mellifera* LBD (c).

(e) Schematic representation of step-by-step construct generation. Restriction sites used for cloning the next fragment into the vector are indicated. The final construct contains honey bee LBD, *D. melanogaster* left and right homology arms (LHA and RHA) with mutated sgRNAs, as well as the *white+* (*w+*) selection marker flanked with inverted terminal repeats and TTAA sequences for its later scarless excision by PiggyBac transposase to restore the reading frame of the nAChR receptor.

Supplementary Figure 4

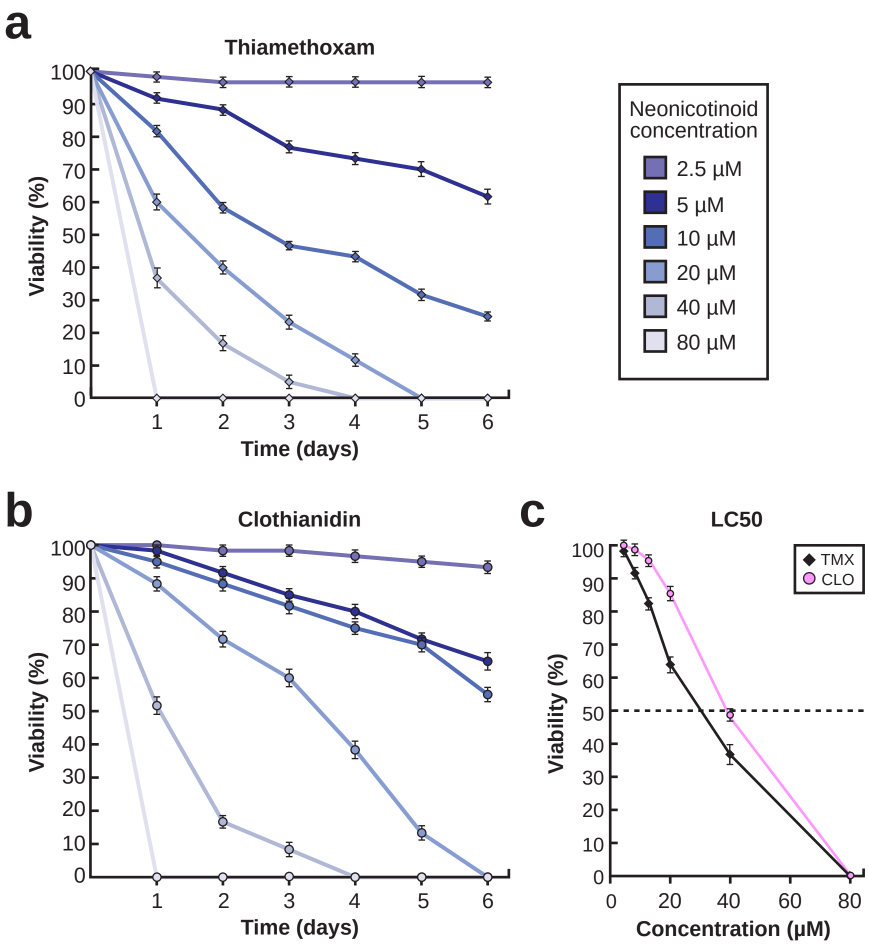

Supplementary Figure 4: Effect of various insecticides concentrations on *Drosophila* viability.

(a-b) Survival curves of WT flies after 6 days of treatment with increasing thiamethoxam and clothianidin concentrations is shown as mean with the standard error from three biological replicates with 20 flies. Viable flies were counted every 24 h.

(c) Viability of WT flies exposed to increasing thiamethoxam (TMX) and clothianidin (CLO) concentrations after 24 h. The dotted line denotes when the survival falls below 50% (LC50).
